# Temporal Screening of High-Risk Food Service Inspections in New York State, 2023–2025: A Case Study Using Multimodal Evidential Learning

**DOI:** 10.3390/foods15111864

**Published:** 2026-05-25

**Authors:** Zi-Heng Cai, Wang-Chin Tsai

**Affiliations:** 1 Graduate School of Design, National Yunlin University of Science & Technology, Yunlin 64002, Taiwan; 2 Department of Creative Design, National Yunlin University of Science & Technology, Yunlin 64002, Taiwan; wangwang@yuntech.edu.tw

**Keywords:** food safety inspection, high-risk screening, evidential deep learning, multimodal learning, uncertainty quantification, risk-based regulation

## Abstract

Food safety inspection systems generate rich historical records, yet converting these records into actionable pre-inspection risk signals remains challenging under limited regulatory resources. The objective of this study was to develop and evaluate a temporally valid, leakage-free, multimodal screening framework for identifying high-risk food service inspections before the upcoming inspection outcome is known. Existing studies have improved inspection prediction with machine learning, but many focus on contemporaneous classification rather than temporally valid high-risk screening, and few jointly model historical numeric behavior, prior narrative context, and predictive uncertainty. To address this gap, this study proposes a temporal high-risk food inspection screening framework based on multimodal evidential learning. Using New York State food service inspection data, we constructed a event-level dataset of 55,454 inspections from 20,082 establishments and predicted whether an upcoming inspection would be high-risk using only pre-inspection information. The proposed evidential deep learning multilayer perceptron integrates current metadata, longitudinal numeric history, and historical inspection comments while producing calibrated uncertainty estimates for selective prediction. On the held-out test set, the proposed model achieved the best overall performance, with an AUROC of 0.846, AUPRC of 0.424, F1 score of 0.431, Brier score of 0.063, and ECE of 0.012, outperforming strong tabular baselines including CatBoost and TabM. Under selective prediction, its retained-set F1 increased from 0.431 at full coverage to 0.542 at 80% coverage. Explainability analysis further showed that predictive gains were driven primarily by historical compliance dynamics, with historical text providing complementary contextual value. These findings support the use of temporally valid, uncertainty-aware multimodal models for risk-based food inspection prioritization.

## 1. Introduction

Foodborne illness remains a major public health challenge, and restaurants continue to be one of the most important settings in which foodborne outbreaks are identified. In the United States, restaurant-associated outbreaks account for a substantial share of reported foodborne disease events, which makes retail food inspection a central component of prevention-oriented public health practice [1]. At the same time, the practical value of inspection systems depends not only on whether inspections are conducted, but also on how inspection information is translated into risk signals that can guide intervention. Early work on restaurant inspection systems already emphasized the need for risk-based grading and prioritization rather than purely uniform inspection routines [2]. However, empirical evidence has also shown that simple inspection scores alone do not always map cleanly onto outbreak occurrence, which suggests that the predictive content of inspection data is more nuanced than a single score can capture [3].

In the present context, the regulated operations of interest are food service and retail food establishments, including restaurants, caterers, delis, banquet facilities, school food-service operations, and other institutions that prepare or serve food directly to consumers. These establishments are important targets for risk-based food control because foodborne illness has substantial public-health consequences. In the United States, the Centers for Disease Control and Prevention estimates that foodborne illness causes approximately 48 million illnesses, 128,000 hospitalizations, and 3000 deaths each year, with norovirus causing the most illnesses and non-typhoidal *Salmonella* causing the most hospitalizations and deaths [4]. Retail food establishments are also a major outbreak setting. For example, CDC surveillance of outbreaks associated with retail food establishments reported 800 outbreaks involving 875 establishments during 2017–2019; among outbreaks with a confirmed or suspected agent, norovirus and *Salmonella* accounted for 47.0% and 18.6% of outbreaks, respectively [5]. The consequences of high-risk food-safety violations extend beyond individual illness events. Serious violations may trigger regulatory enforcement actions, including immediate corrective requirements, suspension of food service, or business closure when an imminent health hazard is present. Under New York State sanitary regulations, an imminent health hazard refers to a violation or condition that makes it probable that food, drink, or continued operation can injure consumers or the public; specified hazards require closure and immediate cessation of food service if they are not corrected at the time of inspection [6]. These legal consequences can create substantial operational disruption for establishments, including remediation costs, lost revenue, staff rescheduling, and potential employment instability during temporary closures. At the societal level, foodborne illness also imposes a large economic burden: recent USDA Economic Research Service estimates place the annual cost of foodborne illness in the United States at approximately $74.7 billion in 2023 dollars [7]. These public-health, regulatory, and economic consequences underscore the need for inspection systems that can identify high-risk establishments before severe violations are observed during the current inspection.

A second line of research has shown that information disclosure can change both regulatory performance and market behavior. The introduction of public grading systems for restaurant hygiene has been associated with measurable improvements in hygiene quality, consumer response, and foodborne illness outcomes [8,9]. More recent evidence indicates that publicly accessible disclosure systems can improve inspection scores and that point-of-service grading and posting practices are associated with lower foodborne outbreak rates at the jurisdictional level [10,11]. These findings support the idea that inspection data are not merely administrative records; they can also function as actionable signals for both regulators and the public. Nevertheless, disclosure-based systems are primarily designed to communicate current or recent inspection outcomes, and they do not by themselves solve the problem of how to identify high-risk inspections before the next event occurs.

Related studies also suggest that the most informative components of inspection history are often more specific than overall scores. Work on inspection-based risk assessment has shown that critical and repeat violations can provide a more meaningful representation of food safety risk than undifferentiated score totals [12]. Likewise, item-level comparisons between outbreak and non-outbreak restaurants have shown that certain inspection criteria are more strongly associated with outbreak settings than others [13]. Recent studies from Finland further indicate that routine inspection results and official grading patterns can be linked to foodborne outbreaks and regional foodborne disease incidence, reinforcing the view that inspection histories contain prospectively useful epidemiological information when analyzed appropriately [14,15]. These studies suggest that food safety risk is better understood as a longitudinal and structured process rather than as a one-time inspection outcome.

The main weakness of current inspection systems is therefore not the absence of inspection records, but the limited ability of routine scores, disclosure-oriented systems, and retrospective analyses to transform historical inspection information into prospective, case-level risk signals before the next inspection occurs. Several analytical strategies have been used to study temporal patterns in food inspection and food-safety surveillance. Rule-based or score-based approaches summarize recent inspection outcomes using violation counts, grades, or repeat-violation indicators; epidemiological studies examine associations between inspection histories and outbreaks; and machine-learning approaches use structured inspection records, complaint data, or engineered lag features to predict inspection outcomes [12,13,14,15,16,17]. These approaches have generated valuable evidence, but they also have important limitations for prospective inspection prioritization. Score-based summaries can compress complex longitudinal and narrative information into a small number of indicators. Retrospective outbreak-association studies are informative for explaining past events but are less directly suited to pre-inspection screening. Random-split machine-learning evaluations can overestimate real-world performance when future events or establishment histories are indirectly mixed with past data. Conventional classifiers also tend to provide point predictions without explicitly representing whether a prediction is reliable or uncertain. These limitations motivate a temporally ordered modeling strategy that uses only pre-inspection information, integrates structured and textual histories, and reports predictive uncertainty for operational triage. Accordingly, this study was guided by three research hypotheses. First, a leakage-free temporal framework using only pre-inspection information can identify high-risk food service inspections more effectively than models based primarily on static metadata. Second, historical numeric compliance patterns and prior inspection comments provide complementary information, such that multimodal fusion improves high-risk screening compared with using either metadata or structured history alone. Third, an evidential learning framework can improve probabilistic reliability and support uncertainty-aware selective prediction, allowing high-confidence cases to be prioritized while ambiguous cases are routed for routine or manual review.

Despite this progress, three important gaps remain. First, much of the existing literature evaluates the effects of inspection systems, disclosure practices, or outbreak associations retrospectively, rather than framing the task as prospective high-risk screening. Second, many analyses are based on current-event inspection results, which are informative for explanation but less suitable for true pre-inspection prediction because they risk entangling the target with same-event information. Third, prior narrative information from inspection comments has been used far less systematically than structured scores and counts, even though complaint systems and inspection narratives are increasingly recognized as valuable surveillance inputs in food safety practice [17]. In short, there remains a need for a temporally valid, leakage-free, and multimodally informed framework that can support risk-based inspection prioritization before the current inspection outcome is known.

To address this need, the present study develops a temporal screening framework for predicting high-risk food inspection events using New York State restaurant inspection data. Rather than predicting current inspection grades from same-event information, we formulate the task as prospective identification of high-risk inspections using only pre-inspection information: current metadata, longitudinal numeric history, and historical inspection comments from prior events. We propose an evidential deep learning multilayer perceptron (EDL-MLP) that integrates these structured and textual historical signals while also producing calibrated uncertainty estimates for selective prediction and triage. The contribution of this study is threefold. First, it reframes food inspection prediction as a forward-looking high-risk screening problem rather than a contemporaneous classification task. Second, it introduces a leakage-free multimodal framework that combines historical numeric behavior with prior narrative context. Third, it shows that improved discrimination, calibration, interpretability, and uncertainty-aware prioritization can be achieved within a single regulatory decision-support framework. The remainder of the paper presents the data and methods, reports the empirical results, and then discusses their implications for risk-based food safety control.

## 2. Methodology

### 2.1. Study Design and Data Source

This study used the active New York State Food Service Establishment Inspections archive from Health Data NY and restricted the analytic window to inspection events observed from 2023 to 2025 [18]. The public archive can be consulted through Health Data NY as “Food Service Establishment Inspections: Beginning 2005 (ACTIVE)” at https://health.data.ny.gov/ (accessed on 10 March 2026). Although the official archive contains records beginning in 2005, the present analysis focused on inspections conducted from 2023 to 2025. This window was selected to provide a recent and policy-relevant inspection period, avoid combining contemporary records with much older inspection environments, and support a temporally ordered model-development design. The resulting sample included 55,454 inspection events from 20,082 food service operations, which provided sufficient event-level observations for training, validation, and future-period testing. Therefore, the 2023–2025 window is sufficient for the objective of this study: evaluating short-horizon, leakage-free, temporal screening of high-risk inspections in a contemporary regulatory setting. At the same time, the study does not claim to characterize all long-term food inspection trends since 2005; longer historical windows and external jurisdictions remain important directions for future validation. The raw archive is recorded at the violation-row level, so a single inspection may appear in multiple rows. We therefore constructed the analytic sample in three steps. First, we extracted the required fields from the official archive. Second, we aggregated violation rows into inspection events. Third, we built a leakage-free modeling table for temporal prediction.

**Figure 1:**
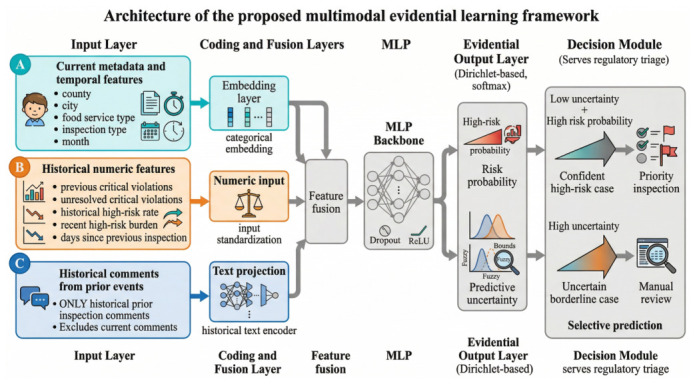
Overall architecture of the proposed multimodal evidential learning framework.

Figure 1 presents the overall architecture of the proposed multimodal evidential learning framework. The model integrates current metadata and temporal features, historical numeric compliance features, and historical comments from prior events. These inputs are encoded and fused through an MLP backbone with a Dirichlet-based evidential output layer, which produces both high-risk probabilities and predictive uncertainty. The final decision module supports regulatory triage by distinguishing confident high-risk cases from uncertain borderline cases.

The final sample contained 154,858 raw rows, which were aggregated into 55,454 inspection events from 20,082 unique establishments. Let *j* index establishments and let *t* index inspections within establishment *j* in chronological order. Each inspection event was uniquely identified by

(1) $$(oid_{j},day_{jt},typ_{jt}),$$

where $oid_{j}$ denotes the official establishment identifier, $day_{jt}$ denotes the inspection date, and $typ_{jt}$ denotes the inspection type.

### 2.2. Event Construction and Outcome Definition

For each establishment *j* at inspection time *t*, let $cri_{jt}$ denote the number of critical violations, let $unc_{jt}$ denote the number of unresolved critical violations, and let $non_{jt}$ denote the number of noncritical violations recorded for that event.

The primary outcome was a binary high-risk label:

(2) $$rsk_{jt}=\left\{\begin{array}{cc} 1, & \mathrm{if}\;cri_{jt}\geq 2\;\mathrm{or}\;unc_{jt}>0,\\ 0, & \mathrm{otherwise}. \end{array}\right.$$

This definition operationalizes regulatory priority by treating events with multiple critical violations or any unresolved critical violation as higher-risk inspections. In addition, we defined an auxiliary indicator

(3) $$any_{jt}=I\left(cri_{jt}\geq 1\right),$$

which was used only for historical feature construction and descriptive analyses.

Importantly, the current-event variables $cri_{jt}$, $unc_{jt}$, $non_{jt}$, $any_{jt}$, and $rsk_{jt}$ were used to define outcomes and historical summaries, but were never used as predictors for the same inspection event.

### 2.3. Leakage-Free Predictor Design

Let i=1,…,N index inspection events in the final modeling table. For each event *i*, the predictor vector was constructed as

(4) $$x_{i}=\left[cat_{i};num_{i};tvec_{i}\right],$$

where $cat_{i}$ is the categorical block, $num_{i}$ is the numeric block, and $tvec_{i}$ is the leakage-free historical text embedding.

All predictors were restricted to information available no later than the start of the current inspection. Current-event violation counts and current-event comments were excluded from model input.

#### 2.3.1. Current Metadata and Calendar Features

The categorical block contained five variables:

(5) $$cat_{i}=\left(cty_{i},cit_{i},ftyp_{i},fdes_{i},typ_{i}\right),$$

where $cty_{i}$ is county, $cit_{i}$ is city, $ftyp_{i}$ is food service type, $fdes_{i}$ is food service description, and $typ_{i}$ is inspection type.

The calendar block included inspection year, inspection month, and a binary re-inspection flag:

(6) $$year_{i},\;mon_{i},\;rin_{i}.$$

To capture annual seasonality smoothly, month was encoded by

(7) $$msin_{i}=\sin\left(\frac{2\pi \;mon_{i}}{12}\right),\;mcos_{i}=\cos\left(\frac{2\pi \;mon_{i}}{12}\right).$$

#### 2.3.2. Historical Numeric Features

For each establishment, historical features were computed using only inspections prior to the current event.

##### Inspection Count and Elapsed Time

Let

(8) $$prv_{jt}=\sum_{\tau <t}1$$

denote the number of prior inspections observed for establishment *j* before time *t*. The elapsed time since the previous inspection was defined as

(9) $$gap_{jt}=\left\{\begin{array}{cc} day_{jt}-day_{j,t-1}, & \mathrm{if}\;prv_{jt}>0,\\ 0, & \mathrm{if}\;prv_{jt}=0. \end{array}\right.$$

##### One-Step Lag Features

For the immediately previous inspection, we defined

(10) $$pcri_{jt}=cri_{j,t-1},\;punc_{jt}=unc_{j,t-1},\;pnon_{jt}=non_{j,t-1},$$

(11) $$pany_{jt}=any_{j,t-1},\;prsk_{jt}=rsk_{j,t-1},\;prin_{jt}=rin_{j,t-1},$$

whenever $prv_{jt}>0$. For first-observed inspections, these lag features were set to zero.

##### Historical Mean Features

Long-run historical averages were computed as

(12) $$acri_{jt}=\left\{\begin{array}{cc} \frac{1}{prv_{jt}}\sum_{\tau <t}cri_{j\tau }, & prv_{jt}>0,\\ 0, & prv_{jt}=0, \end{array}\right.$$

(13) $$aunc_{jt}=\left\{\begin{array}{cc} \frac{1}{prv_{jt}}\sum_{\tau <t}unc_{j\tau }, & prv_{jt}>0,\\ 0, & prv_{jt}=0, \end{array}\right.$$

(14) $$anon_{jt}=\left\{\begin{array}{cc} \frac{1}{prv_{jt}}\sum_{\tau <t}non_{j\tau }, & prv_{jt}>0,\\ 0, & prv_{jt}=0, \end{array}\right.$$

(15) $$aany_{jt}=\left\{\begin{array}{cc} \frac{1}{prv_{jt}}\sum_{\tau <t}any_{j\tau }, & prv_{jt}>0,\\ 0, & prv_{jt}=0, \end{array}\right.$$

(16) $$arsk_{jt}=\left\{\begin{array}{cc} \frac{1}{prv_{jt}}\sum_{\tau <t}rsk_{j\tau }, & prv_{jt}>0,\\ 0, & prv_{jt}=0, \end{array}\right.$$

(17) $$arin_{jt}=\left\{\begin{array}{cc} \frac{1}{prv_{jt}}\sum_{\tau <t}rin_{j\tau }, & prv_{jt}>0,\\ 0, & prv_{jt}=0. \end{array}\right.$$

##### Recent Rolling Features

To capture short-run recurrence, we computed rolling sums over the most recent three prior inspections. Let

(18) $$k_{jt}=\min\left(3,prv_{jt}\right).$$

Then

(19) $$lcri_{jt}=\sum_{s=1}^{k_{jt}}cri_{j,t-s},\;lnon_{jt}=\sum_{s=1}^{k_{jt}}non_{j,t-s},\;lrsk_{jt}=\sum_{s=1}^{k_{jt}}rsk_{j,t-s}.$$

When $prv_{jt}=0$, these rolling features were set to zero.

Collectively, the numeric block was

(20) $$\begin{array}{cc} num_{i}= & (year_{i},mon_{i},msin_{i},mcos_{i},rin_{i},prv_{i},gap_{i},\\ \; & \;\;pcri_{i},punc_{i},pnon_{i},pany_{i},prsk_{i},prin_{i},\\ \; & \;\;acri_{i},aunc_{i},anon_{i},aany_{i},arsk_{i},arin_{i},\\ \; & \;\;lcri_{i},lnon_{i},lrsk_{i}). \end{array}$$

#### 2.3.3. Leakage-Free Historical Text Features

Let $com_{jt}$ denote the free-text inspection comment recorded for establishment *j* at inspection time *t*. To prevent target leakage, $com_{jt}$ from the current event was never used to predict $rsk_{jt}$. Instead, we constructed a historical text sequence using only prior comments.

After lowercasing and whitespace normalization, the historical text string for event (j,t) was defined as

(21) $$htxt_{jt}=\mathrm{join}\left(com_{j,t-1},\ldots ,com_{j,t-k_{jt}}\right),$$

where $k_{jt}=\min\left(3,prv_{jt}\right)$. If no prior inspection was available, $htxt_{jt}$ was set to the empty string.

The historical text string was converted to a dense semantic vector using the pretrained sentence encoder sentence-transformers/all-MiniLM-L6-v2, which is based on the Sentence-BERT framework and produces 384-dimensional sentence embeddings [19,20]. Accordingly, in this study,

(22) $$tvec_{jt}=enc\left(htxt_{jt}\right)\in R^{384},$$

where enc(·) denotes the frozen pretrained sentence encoder. The encoder was not fine-tuned on the current prediction task; only the downstream text-projection layer and the MLP-based prediction model were optimized using the training data. We selected all-MiniLM-L6-v2 because the inspection comments were generally short narrative records, and this encoder provides compact sentence-level semantic representations with favorable computational efficiency. Its 384-dimensional output also allowed the historical text branch to be fused with structured tabular features without introducing an excessively high-dimensional text representation. This design was intended to capture prior narrative context while reducing task-specific overfitting and preserving the leakage-free setting, because only comments from inspections prior to the current event were encoded.

### 2.4. Temporal Split and Model Selection

To mimic prospective deployment, we used a strict chronological split rather than a random split. The cut points were chosen before model fitting and were intended to create a realistic model-development sequence: an earlier training period, a subsequent validation period for model and threshold selection, and a future held-out test period for final evaluation. Specifically, inspections before 1 July 2024 were assigned to the training set because this period provided the largest historical development sample from which the models could learn establishment-level compliance patterns. The period from 1 July 2024 to 31 December 2024 was used as a contiguous six-month validation window immediately preceding the test period. This validation window was used for hyperparameter selection, classification-threshold selection, and uncertainty-threshold selection, while still ensuring that no information from 2025 entered model development. Finally, inspections from 1 January 2025 onward were held out as the test set, representing future inspections under a prospective screening scenario. This design avoided random-split leakage, preserved the temporal ordering of inspection histories, and ensured that all reported test-set results reflected performance on inspections occurring after the model-development period. Let trn, val, and tes denote the training, validation, and test sets, respectively:

(23) $$trn=\{i:day_{i}<2024-07-01\},$$

(24) $$val=\{i:2024-07-01\leq day_{i}\leq 2024-12-31\},$$

(25) $$tes=\{i:day_{i}\geq 2025-01-01\}.$$

All preprocessing steps were fit using training data only. Numerical variables were standardized with training-set means and standard deviations. For neural models, categorical variables were index-encoded and mapped to learned embeddings, with unseen levels assigned to an unk token. For linear and non-CatBoost tree models, categorical variables were one-hot encoded using training-set levels only. CatBoost used native categorical handling.

Hyperparameters were selected on the validation set, and the primary model-selection metric was validation AUPRC because the positive class was expected to be less prevalent than the negative class. After the best hyperparameter setting was chosen, the classification threshold was selected on the validation set by maximizing the F1 score.

### 2.5. Proposed Model

#### 2.5.1. Overview

The proposed model is an evidential multilayer perceptron with multimodal fusion, denoted as EDL-MLP. It combines three information sources: categorical metadata, numeric history features, and leakage-free historical text embeddings.

#### 2.5.2. Categorical Embedding Block

Let the categorical input contain m=5 fields. For field *m*, let $c_{im}$ denote the category index for event *i*. Each field was mapped to an embedding vector:

(26) $$cem_{im}=emb_{m}\left(c_{im}\right)\in R^{d_{m}},\;m=1,\ldots ,5.$$

#### 2.5.3. Historical Text Projection

The historical text embedding $tvec_{i}$ was projected to a compact task-specific representation:

(27) $$gtxt_{i}=\mathrm{relu}\left(wtxt\;tvec_{i}+btxt\right).$$

#### 2.5.4. Feature Fusion and MLP Backbone

The fused input representation was

(28) $$fuse_{i}=\left[cem_{i1};cem_{i2};cem_{i3};cem_{i4};cem_{i5};num_{i};gtxt_{i}\right].$$

This vector was then passed through an *L*-layer multilayer perceptron:

(29) $$h_{i}^{\left(1\right)}=\mathrm{relu}\left(w^{\left(1\right)}fuse_{i}+b^{\left(1\right)}\right),$$

(30) $$h_{i}^{\left(\ell \right)}=\mathrm{relu}\left(w^{\left(\ell \right)}h_{i}^{\left(\ell -1\right)}+b^{\left(\ell \right)}\right),\;\ell =2,\ldots ,L.$$

Dropout was applied between hidden layers to reduce overfitting. The hidden width, depth, dropout rate, embedding sizes, and text projection size were tuned on the validation set.

#### 2.5.5. Evidential Output Layer

Instead of using a standard sigmoid or softmax output, the model produced nonnegative evidence for the two classes:

(31) $$out_{i}=w^{\left(o\right)}h_{i}^{\left(L\right)}+b^{\left(o\right)},$$

(32) $$evid_{ik}=\mathrm{softplus}\left(out_{ik}\right),\;k\in \left\{0,1\right\}.$$

The evidence was converted into Dirichlet concentration parameters:

(33) $$alfa_{ik}=evid_{ik}+1,$$

and their sum was

(34) $$sumi_{i}=\sum_{k=0}^{1}alfa_{ik}.$$

The predictive class probabilities were defined by the Dirichlet posterior mean:

(35) $$prob_{ik}=\frac{alfa_{ik}}{sumi_{i}},\;k\in \left\{0,1\right\}.$$

The final binary decision rule was

(36) $${\hat{rsk}}_{i}=I\left(prob_{i1}\geq thrs\right),$$

where thrs is the validation-selected threshold.

### 2.6. Training Objective

Let

(37) $$lab_{i}=\left(lab_{i0},lab_{i1}\right)$$

denote the one-hot encoded label vector for event *i*.

#### 2.6.1. Data-Fit Term

The expected squared-error term was

(38) $$lerr_{i}=\sum_{k=0}^{1}{\left(lab_{ik}-prob_{ik}\right)}^{2}.$$

#### 2.6.2. Variance Term

The predictive variance term was

(39) $$lvar_{i}=\sum_{k=0}^{1}\frac{alfa_{ik}\left(sumi_{i}-alfa_{ik}\right)}{sumi_{i}^{2}\left(sumi_{i}+1\right)}.$$

#### 2.6.3. Regularization Term

To penalize unsupported evidence for the wrong class, we defined adjusted concentration parameters

(40) $$talf_{ik}=lab_{ik}+\left(1-lab_{ik}\right)alfa_{ik},$$

and the regularization term

(41) $$lkl_{i}=\mathrm{KL}\left[\mathrm{Dir}\left(talf_{i}\right)\;∥\;\mathrm{Dir}\left(ones\right)\right],$$

where ones=(1,1).

#### 2.6.4. Overall Loss

The epoch-dependent annealing weight was

(42) $$lamt=\min\left(1,\frac{epo}{tann}\right),$$

where epo is the current epoch and tann is the annealing horizon.

The minibatch loss was

(43) $$loss=\frac{1}{bsiz}\sum_{i=1}^{bsiz}\left(lerr_{i}+lvar_{i}+lamt\;lkl_{i}\right).$$

The model was optimized with AdamW using mini-batch training and early stopping based on validation AUPRC.

### 2.7. Implementation and Hyperparameter Tuning

The proposed EDL-MLP was implemented in Python 3.10 using a PyTorch-based training pipeline. Hyperparameters were selected on the validation set, and the primary model-selection criterion was validation AUPRC, consistent with the imbalanced high-risk screening objective. The classification threshold was selected after model training on the validation set by maximizing the F1 score over candidate probability thresholds from 0.05 to 0.95 with a step size of 0.01. This selected threshold was then fixed and applied unchanged to the held-out test set. To address class imbalance during neural-network training, mini-batches were sampled using inverse-frequency class weights computed from the training set. Early stopping was based on validation AUPRC. Table 1 reports the tuning ranges and final selected values for the proposed model.

### 2.8. Uncertainty Quantification and Selective Prediction

A key advantage of evidential learning is that predictive uncertainty is derived directly from the total evidence mass. For binary classification, the uncertainty score was defined as

(44) $$unc_{i}=\frac{2}{sumi_{i}}.$$

A larger $unc_{i}$ indicates lower confidence.

To evaluate selective prediction, we first obtained the uncertainty threshold corresponding to a target coverage level cov from the validation set:

(45) $$qcov=Q_{cov}\left(\{unc_{i}:i\in val\}\right),$$

where $Q_{cov}\left(\cdot \right)$ denotes the empirical cov-quantile.

We then defined the retained subset in the test set as

(46) $$sel\left(cov\right)=\left\{i\in tes:unc_{i}\leq qcov\right\}.$$

Performance at coverage level cov was evaluated on the retained subset only. In particular, we report retained-set F1 scores at cov=0.90 and cov=0.80.

### 2.9. Multimodal Explainability Analysis

To interpret the proposed EDL-MLP after model training, we conducted a post hoc multimodal explainability analysis using SHAP additive attributions [21]. SHAP values were computed with the DeepExplainer implementation for the trained PyTorch 2.1.0. model. The explained model output was the predicted probability of the high-risk class, $prob_{i1}$, defined by the Dirichlet posterior mean in Equation (35). The background set for SHAP estimation consisted of 512 inspection events sampled from the training set while preserving the approximate positive-class prevalence of the training data. Global explanations were then computed on the held-out test set. The explainer was applied to the fused model representation rather than to the raw text strings or unprocessed categorical labels. Specifically, explanations were computed after categorical variables had been mapped to embedding vectors, numerical variables had been standardized using training-set statistics, and historical text embeddings had been projected through the text-projection layer. Thus, the initial SHAP values were defined over the fused input components in Equation (28). For interpretability, these fused-input attributions were subsequently aggregated back to raw fields and modality groups before being reported.

Let ${\hat{p}}_{i}=prob_{i1}$ denote the predicted probability of the high-risk class for event *i*. For each event, the local explanation is written as

(47) $${\hat{p}}_{i}=base+\sum_{k=1}^{K}shap_{ik},$$

where base is the expected model output over the SHAP background sample, and $shap_{ik}$ is the attribution of fused input component or aggregated feature group *k* to event *i*.

#### 2.9.1. Global Feature Importance

To quantify the overall contribution of each feature, we computed the mean absolute attribution:

(48) $$fimp_{k}=\frac{1}{N}\sum_{i=1}^{N}\left|shap_{ik}\right|,$$

where larger values indicate stronger average influence on the model output. These global importance scores were used to rank the top predictors in the test set.

#### 2.9.2. Grouped Modality Contribution

Because the proposed model fuses structured metadata, historical numeric variables, and leakage-free historical text representations, we further summarized attributions at the modality level. Let g∈{meta,hist,text} denote a modality group, and let $G_{g}$ be the set of input components associated with that group. The grouped contribution for event *i* was defined as

(49) $$gcon_{ig}=\sum_{k\in G_{g}}shap_{ik},$$

and the average grouped importance was

(50) $$gimp_{g}=\frac{1}{N}\sum_{i=1}^{N}\left|gcon_{ig}\right|.$$

This grouping allowed us to compare the relative explanatory roles of current metadata and temporal features, historical numeric behavior, and historical text information.

#### 2.9.3. Aggregation Rule for Embedded and Projected Inputs

Because the proposed model contains embedded categorical fields and a projected historical-text representation, SHAP values were first obtained at the fused-input level and then aggregated for reporting. For each categorical field, SHAP values across all coordinates of its embedding vector were summed and reported as the contribution of the corresponding raw categorical variable. Each standardized numerical variable retained its own feature-level attribution. For the historical-text branch, SHAP values across all coordinates of the projected text representation $gtxt_{i}$ were summed and reported as a single historical-text contribution. This aggregation strategy avoids over-interpreting individual embedding or projection coordinates while preserving the ability to compare current metadata, historical numerical behavior, and historical narrative context. The global ranking is therefore based on aggregated field-level attributions, whereas the modality comparison is based on grouped attributions for metadata and temporal variables, historical numeric variables, and historical text.

#### 2.9.4. Case-Level Analysis

To complement the global analysis, we selected two representative test cases for local explanation. The first set was defined as

(51) $$seta=\{i:y_{i}=1,\;{\hat{rsk}}_{i}=1,\;unc_{i}\leq qlo,\;prob_{i1}\geq 0.80\},$$

where qlo denotes a low-uncertainty cutoff estimated from the test-set uncertainty distribution. A representative event from seta was used as a low-uncertainty true high-risk case.

The second set was defined as

(52) $$setb=\{i:unc_{i}\geq qhi,\;|prob_{i1}-thrs|\leq del\},$$

where qhi denotes a high-uncertainty cutoff and del is a small margin around the classification threshold. A representative event from setb was used as an ambiguous high-uncertainty case.

For both cases, we visualized local attributions using waterfall-style decomposition and summarized the predicted high-risk probability and predictive uncertainty. When discussing the historical text branch, only pre-inspection historical comments were quoted; current-event comments were never used as model input and explanation evidence.

### 2.10. Baseline Models

The baseline set covered linear, tree-based, and deep tabular learners:

(53) {logit,rf,xgb,lgbm,catb,mlp,ftt,tabm}.

Specifically, logit denotes logistic regression; rf denotes random forest; xgb denotes XGBoost [22]; lgbm denotes LightGBM [23]; catb denotes CatBoost [24]; mlp denotes a standard multilayer perceptron; ftt denotes FT-Transformer [25]; and tabm denotes TabM [26].

All baselines used the same temporal split and the same leakage-free predictors. For models that did not natively consume text, the historical text input entered as the dense vector $tvec_{i}$. For CatBoost, raw categorical fields were passed natively while $num_{i}$ and $tvec_{i}$ were treated as continuous features. For linear, tree-based, and non-evidential neural models, probability thresholds were selected on the validation set using the same rule as the proposed model.

### 2.11. Ablation Design

Ablation experiments were reported separately from the main baseline comparison. Four targeted ablations were conducted. First, we removed the evidential head and replaced it with a standard sigmoid output trained by weighted binary cross-entropy, while keeping the same multimodal backbone. This variant isolates the contribution of evidential learning. Second, we removed the historical text branch by excluding $tvec_{i}$ from the fused input. This variant tests whether leakage-free historical comments provide information beyond structured history features. Third, we removed all history-based numeric variables and retained only current metadata and calendar features. This variant evaluates the predictive value of inspection history. Fourth, we removed the recent rolling features $\left(lcri_{i},lnon_{i},lrsk_{i}\right)$ while retaining one-step lag features and long-run historical averages. This variant isolates the added value of short-run recurrence information.

### 2.12. Evaluation Metrics

All models were evaluated on the held-out test set under the same temporal split. The primary discrimination metrics were AUROC, AUPRC, precision, recall, and F1 score. AUROC was interpreted on a scale from 0.5 to 1.0, where 0.5 indicates chance-level discrimination and 1.0 indicates perfect discrimination. AUPRC was interpreted relative to the positive-class prevalence, with higher values indicating better ranking of rare high-risk events and 1.0 indicating perfect precision–recall performance. Precision, recall, and F1 range from 0 to 1, with higher values indicating better threshold-based screening performance. Because the task was an imbalanced binary screening problem, AUPRC and recall were treated as particularly important summary measures. Probabilistic reliability was evaluated using the Brier score and expected calibration error (ECE). Both measures range from 0 to 1 in binary probabilistic prediction, with lower values indicating better reliability. A Brier score closer to 0 indicates smaller mean squared error between predicted probabilities and observed outcomes, while an ECE closer to 0 indicates better agreement between predicted probabilities and empirical event frequencies. ECE was computed from ten equal-frequency bins on test-set predicted probabilities. Several metrics were reported because high-risk inspection screening has multiple operational objectives. AUROC and AUPRC summarize ranking quality, precision reflects the proportion of predicted high-risk inspections that are truly high-risk, recall reflects the proportion of true high-risk inspections captured by the model, and F1 summarizes the balance between precision and recall at the selected threshold. Calibration metrics are also necessary because a regulatory triage model should provide reliable probabilities rather than only binary classifications. Therefore, the use of multiple indicators provides a more complete and transparent assessment of discrimination, screening utility, and probabilistic reliability than any single best-performing metric alone.

For uncertainty-aware decision support, we additionally reported retained-set performance under selective prediction. In particular, we evaluated F1 at 90% coverage and 80% coverage, together with the corresponding retained recall. All point estimates reported in the Results section were computed on the test set only after model development, hyperparameter tuning, threshold selection, and uncertainty-threshold selection had been completed using the training and validation sets.

**Figure 2:**
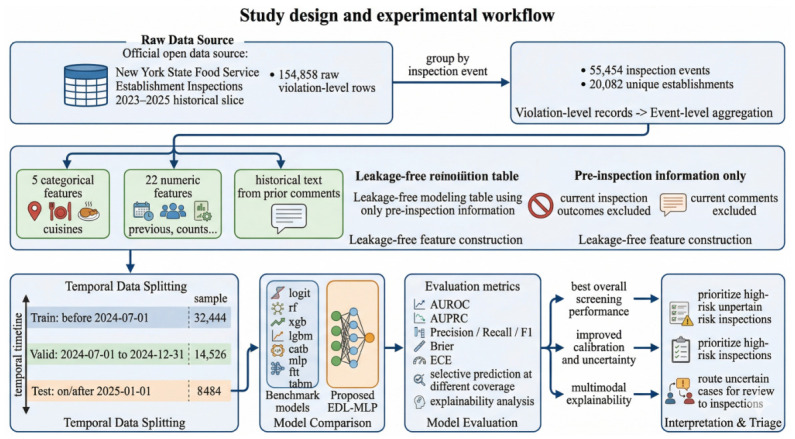
Study design and experimental workflow.

To summarize the overall study design and experimental pipeline, Figure 2 provides an integrated overview of the data processing, leakage-free feature construction, temporal splitting, model comparison, and evaluation workflow used in this study. The official raw inspection archive was first aggregated from violation-level records to event-level observations. A leakage-free modeling table was then constructed using only pre-inspection information, including categorical metadata, historical numeric features, and historical text from prior comments. The resulting dataset was temporally split into training, validation, and test sets and used for benchmark comparison, model evaluation, and interpretation-oriented triage analysis.

## 3. Results: Temporal Screening Analysis and Model Evaluation

The Results section is organized to reflect the two aims of the study. First, we describe the temporal structure of the New York State food service inspection case study, including sample composition, temporal drift, inspection-history depth, and the practical implications of applying a screening model to future inspection periods. Second, we evaluate the proposed multimodal evidential learning framework against baseline models, ablation variants, uncertainty-aware triage strategies, and explainability analyses. This structure is intended to present the temporality analysis and the method-evaluation results as complementary components of the same prospective high-risk inspection screening problem.

### 3.1. Sample Profile and Temporal Drift

The final analytic sample contained 55,454 inspection events from 20,082 unique food service operations (Table 2). After applying the chronological split, 32,444 events were assigned to the training set, 14,526 to the validation set, and 8484 to the test set. The prevalence of the high-risk label remained relatively stable across the three periods, ranging from 7.45% in the validation set to 8.31% in the test set, with an overall rate of 7.97%. This pattern indicates that the held-out test period did not exhibit an extreme shift in class prevalence, while still preserving realistic temporal variation. The sample also exhibited clear temporal differences in historical observation depth. The first-visit rate was highest in the training period (52.33%) and declined sharply in the validation and test periods to 16.06% and 9.08%, respectively. This pattern is expected under chronological splitting, because a larger proportion of establishments appear for the first time early in the study window, whereas later periods contain more repeat inspections with accumulated historical records. Consistent with this trend, the median number of prior inspections increased from 0.0 in the training set to 1.0 in the validation set and 2.0 in the test set. The median gap days showed a similar temporal pattern. Because first-visit events were coded as 0 for both prior inspections and gap days, the training set had a median gap of 0.0 days, while the validation and test sets showed substantially longer intervals of 212.5 and 147.0 days, respectively. The uneven history depth across temporal splits has two important implications. Methodologically, this pattern is a direct consequence of chronological evaluation rather than random sampling. Because the study window begins in 2023, many establishments in the early training period are first-observed cases within the available archive, whereas later validation and test periods contain more repeat inspections with accumulated historical records. This does not compromise the fairness of model comparison, because all models were trained, selected, and tested under the same temporal split and were given exactly the same leakage-free information available before each inspection event. No model was allowed to use future records or current-event violation information. However, the pattern does mean that the prediction task becomes more history-informed over time, and models that can effectively use longitudinal compliance information may gain a larger advantage in later periods or among establishments with deeper prior histories. This interpretation is further examined in the ablation and subgroup analyses reported below. Practically, the difference in history depth reflects an important deployment issue for food inspection screening systems. For newly observed establishments or first-visit cases, the model has limited establishment-specific history and must rely mainly on current metadata, calendar features, and inspection-type information. Predictions for such cases should therefore be interpreted as lower-information triage signals and may require routine inspection scheduling or additional manual review. In contrast, for establishments with repeated prior inspections, accumulated numeric histories and prior comments provide richer evidence about compliance behavior, making risk-based prioritization more informative. Thus, the proposed framework is most useful as an adaptive screening tool whose evidential strength increases as inspection histories accumulate, while uncertainty estimates help flag sparse-history or ambiguous cases for cautious interpretation. Re-inspections accounted for 14.46% of the full sample and remained highly stable across the three splits, varying only from 13.79% to 14.86%. This stability suggests that the composition of inspection types remained broadly comparable over time, even though the amount of establishment-specific history increased substantially from train to test. Taken together, the descriptive statistics indicate that the prediction task becomes progressively more history-informed in later periods, which is exactly the type of setting in which a leakage-free temporal framework should be evaluated.

**Figure 3:**
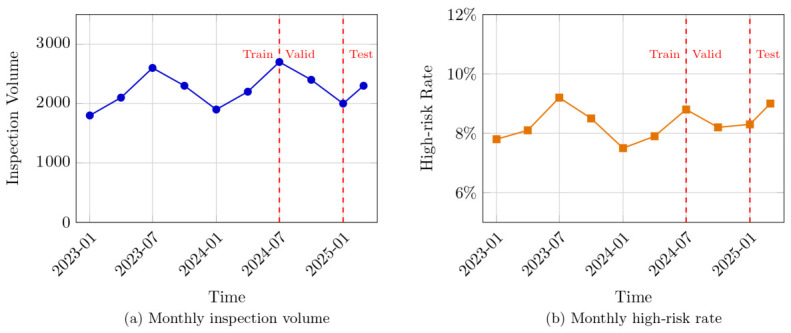
Temporal profile of the study sample. Panel (**a**) shows the monthly inspection volume across the study period, and panel (**b**) shows the monthly prevalence of the high-risk label. Vertical dashed lines mark the chronological boundaries for the training, validation, and test periods.

Figure 3 further visualizes the temporal profile of the study sample. Panel (a) shows that monthly inspection volume fluctuated within a moderate range throughout the study period, without any abrupt collapse or surge around the split boundaries. Panel (b) shows that the monthly prevalence of the high-risk label also varied over time, but remained within a relatively narrow band. These results support two conclusions. First, the study period contains meaningful but not extreme temporal drift, making chronological evaluation substantively necessary. Second, the test period remains comparable enough to the earlier periods to permit a fair assessment of prospective screening performance. Therefore, the results in Table 2 and Figure 3 confirm that the task is neither a static random-split classification problem nor an artificially shifted forecasting exercise, but a realistic temporal risk-screening problem with evolving historical depth and moderate distributional change. From a temporality-analysis perspective, these results show that the screening task contains three distinct temporal features. First, inspection volume and high-risk prevalence varied over the study period, but neither shifted so abruptly that the 2025 test period became incomparable with the development period. Second, establishment-specific history accumulated over time, which made later-period predictions more informative but also created a realistic distinction between first-visit and repeat-inspection cases. Third, the temporal split created a future-period evaluation setting in which model performance reflects prediction after the model-development period rather than random interpolation across mixed years. These features support the interpretation of the study as a temporal high-risk screening case study rather than a conventional static classification exercise.

### 3.2. Overall Benchmark Performance

Table 3 reports the main benchmark comparison on the held-out test set. Overall, the proposed edl-mlp achieved the strongest performance across all evaluation dimensions. It obtained the highest AUROC (0.846), the highest AUPRC (0.424), the highest precision (0.418), the highest recall (0.445), and the highest F1 score (0.431). At the same time, it also yielded the lowest Brier score (0.063) and the lowest expected calibration error (ECE = 0.012), indicating that its advantage was not limited to threshold-dependent classification accuracy, but extended to probabilistic reliability as well. Among the baseline models, catb and tabm were the most competitive. catb achieved an AUPRC of 0.398 and an F1 score of 0.406, while tabm achieved an AUPRC of 0.395 and an F1 score of 0.404. lgbm and xgb also performed strongly, but remained below the two leading baselines. In contrast, logit showed the weakest overall discrimination and screening performance, with an AUPRC of only 0.231 and an F1 score of 0.271. These results indicate that strong nonlinear and ensemble-based learners are clearly preferable to linear screening rules for this task, but the proposed EDL-MLP still provides a further improvement over the best existing tabular baselines. The gain of the proposed model was especially notable in AUPRC, which is the most informative metric for this imbalanced high-risk screening problem. Relative to the strongest baseline, edl-mlp improved AUPRC from 0.398 to 0.424, corresponding to an absolute gain of 0.026. The F1 score improved from 0.406 to 0.431, and the ECE was reduced by more than half, from 0.025 to 0.012. This pattern suggests that the proposed framework delivers a better balance between ranking quality, threshold-based screening performance, and calibration than the competing methods. The AUROC values in Table 3 should be interpreted in light of the task difficulty. The proposed model achieved an AUROC of 0.846, indicating useful discrimination rather than low performance. The more modest AUPRC, precision, recall, and F1 values reflect the prospective, leakage-free, and imbalanced nature of the screening task: the positive class accounted for only 8.31% of the test set, current-event violation information was excluded, and some establishments had limited prior history. Thus, these values reflect the realistic difficulty of predicting future high-risk inspections before the current inspection outcome is known.

**Figure 4:**
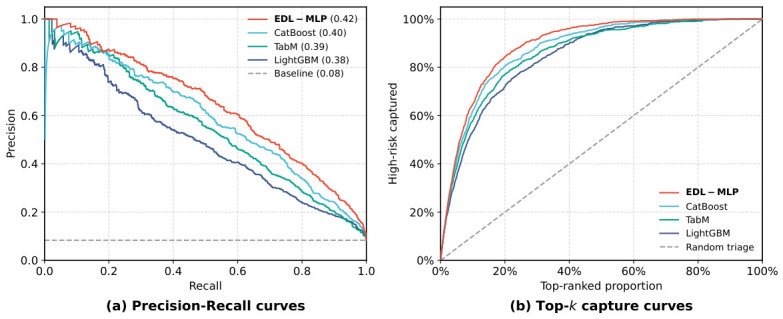
Overall ranking and screening performance of the top models. Panel (**a**) plots the precision–recall curves of the best-performing methods, while panel (**b**) shows the proportion of true high-risk inspections captured when only the top-ranked proportion of events is selected for priority review.

Figure 4a further illustrates the precision–recall trade-off among the top-performing models. Across most of the recall range, the precision–recall curve of edl-mlp remained above those of catb, tabm, and lgbm, confirming that its advantage was not confined to a single operating threshold. Importantly, the gap remained visible in the medium- to high-recall region, which is particularly relevant for regulatory screening settings where missing truly high-risk inspections can be costly. Figure 4b complements the summary statistics in Table 3 by showing the top-*k* capture curves. The proposed model consistently captured a larger proportion of true high-risk inspections when only the top-ranked subset of events was selected for priority review. In other words, when inspection resources are limited and only a fraction of establishments can be prioritized, edl-mlp places a larger share of truly high-risk events near the top of the ranking than the strongest benchmark models. Taken together, Table 3 and Figure 4 show that the proposed method not only performs best under full test-set evaluation, but is also especially well suited to practical risk-based prioritization.

### 3.3. Incremental Value of Historical Numeric and Text Information

Table 4 reports the ablation results of the proposed framework on the held-out test set. The full model achieved the strongest overall performance, with an AUPRC of 0.424, an F1 score of 0.431, a Brier score of 0.063, and an ECE of 0.012. Removing any major component reduced performance, but the magnitude of the decline varied substantially across components, indicating that the gains of the proposed framework were not attributable to a single design choice. The largest performance drop occurred when the historical numeric block was removed. In this setting, AUPRC decreased from 0.424 to 0.318, corresponding to an absolute reduction of 0.106, and F1 decreased from 0.431 to 0.345. The metadata-only variant performed even worse, with an AUPRC of 0.245 and an F1 score of 0.280. These results show that structured historical inspection information is the dominant source of predictive signal in this task. In substantive terms, the model relies heavily on prior high-risk frequency, historical critical-violation burden, unresolved critical issues, and other longitudinal compliance patterns that cannot be recovered from current metadata alone. Leakage-free historical text also provided a meaningful incremental gain. When the historical text branch was removed, AUPRC declined from 0.424 to 0.392, and F1 declined from 0.431 to 0.405. Although this decrease was smaller than the drop caused by removing the historical numeric block, it was still substantial and systematic across metrics. The text ablation also worsened both Brier score and ECE, indicating that historical comments improved not only screening discrimination but also probabilistic reliability. This finding supports the view that prior inspection narratives contain contextual information beyond what can be summarized by counts and binary indicators alone. The recent rolling-history variables contributed additional but more modest gains. Removing these features reduced AUPRC from 0.424 to 0.415 and F1 from 0.431 to 0.422. This pattern suggests that short-run recurrence signals are useful, but that they mainly refine a prediction framework whose core strength already comes from broader historical structure. By contrast, removing the evidential head reduced AUPRC to 0.408 and F1 to 0.415, while ECE deteriorated sharply from 0.012 to 0.041. Thus, the evidential formulation contributed moderate gains in discrimination but much larger gains in calibration, reinforcing the interpretation that its primary value lies in producing more reliable and uncertainty-aware predictions rather than merely shifting threshold-based accuracy.

**Figure 5:**
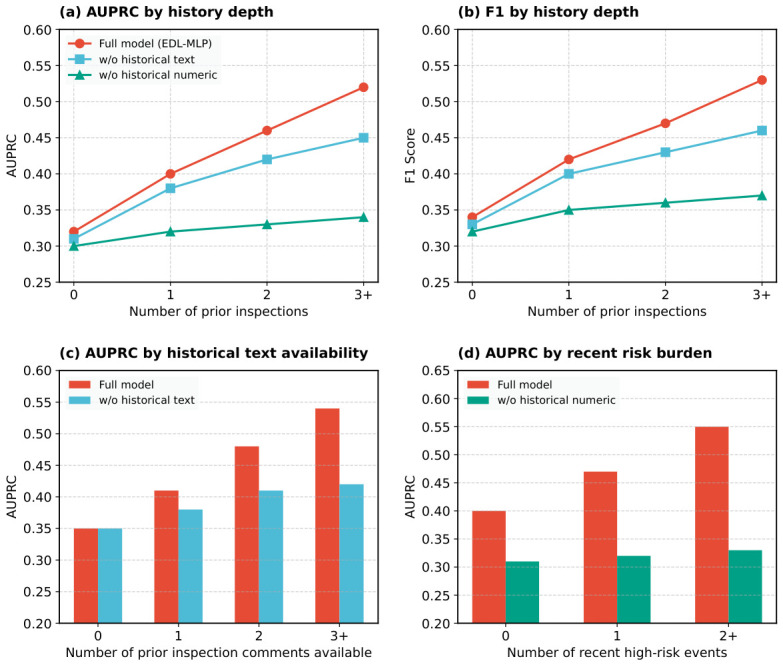
Where the proposed framework gains its advantage. Panel (**a**) compares AUPRC across subgroups defined by prior inspection depth. Panel (**b**) shows the corresponding F1 scores. Panel (**c**) compares subgroup performance by the availability of prior inspection comments, demonstrating that the gap widens as text history increases. Panel (**d**) stratifies performance by recent high-risk burden.

Figure 5 provides a more detailed view of where these gains arise. Panel (a) shows that AUPRC increased monotonically with inspection-history depth for all model variants, but the full model maintained the strongest performance in every subgroup. The gap between the full model and the variant without historical numeric features widened as more prior inspections became available, increasing from a small difference at depth 0 to a much larger difference at depth 3+. Panel (b) shows the same pattern for F1 score. These results indicate that the proposed framework is especially effective when richer establishment history is available, which is consistent with its design as a temporally informed risk-screening model. Panel (c) focuses specifically on the contribution of historical comments. When no prior inspection comment was available, the full model and the no-text variant performed similarly, both at an AUPRC of 0.35. However, once historical comments became available, the performance gap widened steadily. At three or more prior comments, the full model reached an AUPRC of 0.54, compared with 0.42 for the no-text variant. This pattern strongly suggests that the historical text branch extracts useful information only when meaningful prior narrative context exists, which is exactly what would be expected under a leakage-free design. Panel (d) further stratifies performance by recent high-risk burden. As the number of recent high-risk events increased, the AUPRC of the full model rose from 0.40 to 0.55, whereas the variant without historical numeric features remained nearly flat, increasing only from 0.31 to 0.33. This result shows that recent risk recurrence is most effectively captured through structured historical features. Taken together, Table 4 and Figure 5 show that the predictive advantage of the proposed framework is driven primarily by historical numeric behavior, further strengthened by leakage-free historical text, and finally refined by evidential learning and recent recurrence features.

### 3.4. Calibration and Uncertainty-Aware Regulatory Triage

Table 5 evaluates whether the uncertainty estimates produced by the competing models can be used to improve decision quality under selective prediction. At full coverage, edl-mlp already achieved the strongest F1 score (0.431), outperforming catb (0.406) and tabm (0.404). As coverage decreased and only the more confident subset of predictions was retained, the retained-set F1 score increased for all models, but the gain was consistently largest for the proposed method. Specifically, the F1 score of edl-mlp increased from 0.431 at full coverage to 0.486 at 90% coverage and to 0.542 at 80% coverage. By comparison, the corresponding values for catb were 0.406, 0.435, and 0.471, and those for tabm were 0.404, 0.428, and 0.458. This pattern indicates that the uncertainty scores produced by the proposed evidential framework are more effective at separating reliable from unreliable predictions. The relative error reduction at 80% coverage further supports this conclusion. For edl-mlp, the prediction error rate decreased by 19.5%, compared with 10.9% for catb and 9.1% for tabm. Thus, uncertainty-aware triage did not merely improve retained-set performance in an absolute sense; it also yielded a substantially larger practical benefit for the proposed model than for the strongest baselines.

The practical meaning of selective prediction is important. In this study, deferring 10–20% of cases does not mean that these establishments would be ignored, removed from the inspection system, or treated as low risk. Rather, deferral means that the model does not provide an automatic high-confidence prioritization decision for the most uncertain cases. In a real inspection workflow, retained cases could be used for model-assisted priority ranking, whereas deferred cases would remain in the regulatory workflow and could be handled through routine scheduling, inspector review, or additional information collection. Thus, uncertainty-based deferral is best understood as a safeguard against over-reliance on model predictions in borderline cases. Under this interpretation, the 90% coverage setting represents a relatively light-touch triage strategy: approximately the 10% most uncertain cases are routed away from automatic model-assisted prioritization, while the remaining higher-confidence cases are retained for ranking and screening. The 80% coverage setting represents a more conservative strategy, in which approximately the 20% most uncertain cases are deferred for manual or routine review. The retained-set improvements should therefore be interpreted as improvements in the subset of cases for which the model is sufficiently confident, not as a claim that all inspection decisions improve by the same amount. In the present test set, this strategy increased the retained-set F1 score of edl-mlp from 0.431 at full coverage to 0.486 at 90% coverage and 0.542 at 80% coverage, indicating that the uncertainty score can help identify the cases for which model-assisted screening is most reliable.

**Figure 6:**
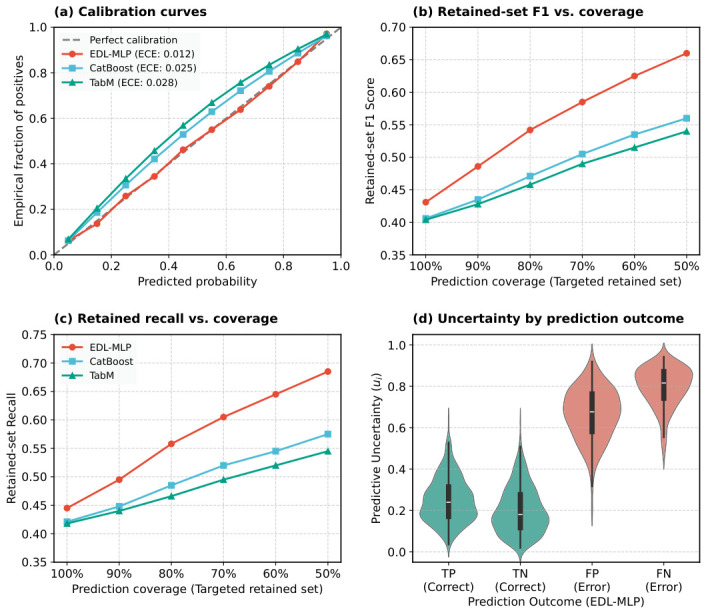
Calibration, selective prediction, and uncertainty structure. Panel (**a**) compares the calibration curves of the proposed model and the strongest baselines. Panel (**b**) shows the retained-set F1 score as coverage decreases. Panel (**c**) presents the retained recall of the high-risk class across corresponding coverage levels, where retained recall is computed within the retained subset only. Panel (**d**) plots the distribution of predictive uncertainty for true positives (TP), true negatives (TN), false positives (FP), and false negatives (FN), showing that predictive errors are associated with markedly higher uncertainty scores.

Figure 6a shows that the proposed model remained closest to the diagonal reference line across most of the probability range, indicating better agreement between predicted probabilities and empirical outcome frequencies. This pattern is consistent with Table 3, where edl-mlp achieved the lowest ECE (0.012), compared with 0.025 for catb and 0.028 for tabm, suggesting that its uncertainty advantage is grounded in better calibration. Figure 6b provides a continuous view of retained-set F1 across coverage levels. As increasingly uncertain cases were deferred, retained-set F1 increased for all models, but the curve of edl-mlp remained consistently above those of the strongest baselines. This indicates that, when attention is restricted to higher-confidence predictions, the proposed model preserves a larger share of useful signal than competing methods. Figure 6c shows the retained recall of the high-risk class within the retained subset. For edl-mlp, retained recall increased from 0.445 at full coverage to 0.495 at 90% coverage and 0.558 at 80% coverage, again exceeding catb and tabm. Because retained recall is computed only within the retained subset, this pattern indicates that uncertainty-based deferral removes a disproportionate share of the more difficult high-risk cases, leaving a more coherent retained subset. Figure 6d links uncertainty directly to prediction outcomes. True positives and true negatives are concentrated at lower uncertainty levels, whereas false positives and false negatives occupy much higher ranges, with false negatives showing the highest uncertainty overall. This confirms that the uncertainty scores are not arbitrary by-products of model fitting, but meaningful indicators of case difficulty and predictive error. Taken together, Table 5 and Figure 6 show that the proposed EDL-MLP not only performs best at full coverage, but also provides better calibration and more actionable uncertainty estimates for resource-aware regulatory triage.

### 3.5. Multimodal Explainability and Case-Level Analysis

**Figure 7:**
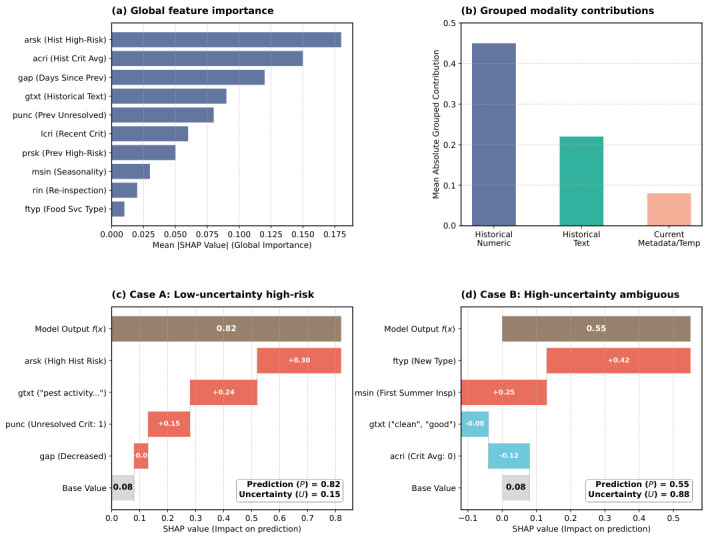
Multimodal explainability and case-level analysis. Panel (**a**) presents the global feature-importance ranking based on mean absolute additive attributions. Panel (**b**) summarizes grouped contribution strength across current metadata and temporal features, historical numeric features, and leakage-free historical text representations. Panel (**c**) shows a representative low-uncertainty true high-risk event (Case A: true high-risk, correctly predicted). Panel (**d**) shows a representative high-uncertainty ambiguous event (Case B: borderline event with high uncertainty). In Panels (**c**,**d**), local attribution values close to 0 indicate little contribution to the case-level prediction; positive values farther from 0 push the prediction toward the high-risk class, whereas negative values farther from 0 push the prediction away from the high-risk class. *P* denotes the predicted high-risk probability, and *U* denotes predictive uncertainty.

To complement the benchmark, ablation, and uncertainty-aware triage results, we further examined how the proposed framework combined structured history and leakage-free historical text. Figure 7 presents both global and local explanations, clarifying why the model performed well overall and why its uncertainty estimates were especially informative in easy versus borderline cases. Figure 7a shows that the most influential predictors were dominated by historical compliance variables rather than static metadata. In particular, arsk, acri, gap, gtxt, and punc ranked among the strongest predictors. This pattern is fully consistent with Table 4, where removing the historical numeric block caused the largest performance drop and removing the historical text branch also produced a clear decline. Figure 7b further shows that historical numeric information had the largest grouped contribution, followed by historical text, whereas current metadata and temporal variables contributed much less on average. Together, these results confirm that the model’s advantage comes primarily from temporally structured evidence, with historical text providing additional contextual information beyond structured summaries. All explanations in Figure 7 were generated using the SHAP procedure described in Section 2.9. The explanations were computed on the fused EDL-MLP representation and then aggregated to interpretable fields and modality groups. Thus, the variable gtxt in Panel (a) represents the total contribution of the projected historical-text branch, rather than an individual word, token, or embedding coordinate. Similarly, the grouped results in Panel (b) summarize the absolute contribution of three pre-specified groups: current metadata and temporal features, historical numeric features, and leakage-free historical text. Panels (c) and (d) use the same aggregation rule for local waterfall-style explanations, so the displayed contributions correspond to interpretable fields or groups rather than raw fused-vector dimensions.

For the local explanations in Figure 7c,d, the magnitude and sign of each attribution should be interpreted relative to 0. A value close to 0 indicates that the feature or feature group contributed little to the case-level prediction. A positive value farther from 0 indicates stronger support for the high-risk class, whereas a negative value farther from 0 indicates stronger support against the high-risk class. The displayed probability *P* is the model-predicted probability of the high-risk class, with values closer to 1 indicating higher predicted risk. The displayed uncertainty *U* is the evidential uncertainty score, with values closer to 0 indicating greater confidence and larger values indicating greater ambiguity or weaker evidence.

Figure 7c presents a representative low-uncertainty true high-risk event (Case A), for which the model assigned a high predicted risk probability (P=0.82) and low uncertainty (U=0.15). The prediction was supported by multiple concordant signals, including high historical risk, adverse historical text, a previous unresolved critical violation, and a shortened inspection gap. In contrast, Figure 7d shows a borderline case with high uncertainty (Case B), where the predicted probability was only slightly above the threshold (P=0.55) and uncertainty was much higher (U=0.88). Here, the attribution pattern was mixed: some features pushed the model toward higher risk, whereas low historical burden and more reassuring text cues pushed in the opposite direction. As a result, the evidence was weaker and internally conflicting. Overall, Figure 7 provides three main insights. First, the predictive strength of the proposed framework is rooted mainly in historical compliance behavior. Second, leakage-free historical text contributes meaningful contextual value beyond structured history. Third, predictive uncertainty is aligned with evidential coherence: low-uncertainty cases show strong and mutually reinforcing signals, whereas high-uncertainty cases show weaker or conflicting evidence. These findings strengthen the interpretation that the proposed EDL-MLP is not only more accurate, but also more transparent and better suited to practical food safety risk screening.

## 4. Discussion and Conclusions

### 4.1. Positioning the Main Findings in the Food Safety Inspection Literature

The present study contributes to the food safety inspection literature in three closely connected ways. First, it extends prior work on inspection-based compliance prediction by moving from a general compliance-classification setting toward a strictly temporal, leakage-free, event-level high-risk screening task. Earlier work has already shown that machine learning can be useful for prioritizing food outlet inspections when open regulatory data are available [16]. At the same time, recent conceptual and comparative studies have emphasized that food safety inspection is not merely a bureaucratic routine, but a structured form of regulatory judgment whose effectiveness depends on how evidence is defined, interpreted, and operationalized in practice [27,28]. Our results build on this line of thinking by showing that a forward-looking risk-screening framework can be both empirically strong and operationally aligned with inspection prioritization.

Second, our findings are consistent with the growing shift toward risk-based inspection strategies. Recent reviews have shown that food business risk is shaped by a combination of establishment history, recurring critical violations, compliance behavior, and contextual operational factors [29]. Comparative work across jurisdictions further suggests that risk-based inspection systems are increasingly differentiated by how they combine fixed regulatory rules with flexible, evidence-based targeting [28]. The present study fits this trajectory well. Rather than attempting to reproduce current inspection outcomes from same-event information, our framework uses only pre-inspection metadata, prior numeric history, and prior narrative history to identify events that warrant elevated attention. In that sense, it is better interpreted as a regulatory screening tool than as a conventional static classifier.

Third, the study aligns with the ongoing digitalization of official food control. Recent evidence from European food control systems shows that digital inspection environments are increasingly used to support consistency, traceability, and analytical reuse of inspection data [30]. Our results suggest that the value of digitalization does not lie only in replacing paper-based workflows, but also in enabling temporally ordered predictive models that can transform archived inspection histories into actionable early-warning signals. This shift from retrospective record keeping to prospective risk screening is, in our view, one of the most important practical implications of data-rich food safety governance.

### 4.2. Why Longitudinal History, Narrative Context, and Calibrated Uncertainty Matter

The empirical pattern observed in this study strongly suggests that longitudinal establishment history is the central predictive substrate for high-risk inspection screening. This interpretation is consistent with broader reviews showing that machine learning in food safety performs best when it can exploit structured, repeatedly measured, and domain-relevant signals rather than relying only on isolated cross-sectional attributes [31,32]. In our results, the historical numeric block produced the largest incremental gain, and the global explanation analysis ranked long-run and recent compliance indicators among the most influential predictors. Conceptually, this makes sense: recurrent high-risk events, unresolved critical problems, and accumulated violation burden are direct behavioral summaries of the inspection process itself.

At the same time, our results also show that structured history is not the full story. The leakage-free historical text branch delivered a smaller but still meaningful gain, and its value became more pronounced as richer narrative history accumulated. This finding is in line with broader discussions of artificial intelligence and big data in the food sector, where the practical value of AI often depends on its ability to incorporate heterogeneous information sources rather than only tabular measurements [33,34]. Narrative inspection comments contain context that is not fully reducible to counts, averages, or binary indicators. They preserve semantic traces of recurring sanitation concerns, pest-related language, operational inconsistency, and inspector-observed patterns that can sharpen risk interpretation when numeric summaries alone remain incomplete.

This also helps explain why explainability was particularly important in our framework. Recent food-related AI studies have repeatedly noted that adoption depends not only on predictive accuracy, but also on the ability to justify why a model reached a given conclusion [35,36]. In our case, the explainability analysis did not merely provide cosmetic transparency; it showed that the model’s strongest evidence came from regulatory meaningful signals, and that the text branch contributed contextual support rather than arbitrary noise. This is important because interpretability in food safety is not only a technical preference, but also a governance requirement whenever models are used to support inspection prioritization.

A second major contribution of this study is the integration of calibrated uncertainty into risk screening. The evidential design improved not only discrimination but also calibration, and the selective-prediction results showed that removing the most uncertain cases yielded a larger retained-set gain for the proposed model than for strong baselines. This pattern is consistent with the broader literature showing that uncertainty estimation becomes most useful when it is tied to trustworthy probability outputs rather than appended post hoc to poorly calibrated predictors [37,38]. In our results, low-uncertainty cases were characterized by coherent and mutually reinforcing evidence, whereas high-uncertainty cases tended to display mixed or conflicting signals. This suggests that uncertainty is functioning here as a substantive indicator of evidential ambiguity, not simply as a numerical side product. This interpretation is also consistent with recent agricultural machine-learning work emphasizing that uncertainty-aware representations can improve robustness and practical decision support. For example, recent work on fuzzy-augmented machine-learning models for livestock feed efficiency and disease-status prediction suggests that fuzzy membership representations can help capture nonlinear relationships and reduce decision-boundary ambiguity in data-driven agricultural systems [39]. They share an important practical motivation: predictions used in operational food or agricultural decision systems should not only be accurate, but should also express the degree of confidence or ambiguity associated with each decision. In the present study, the Dirichlet evidential output layer serves this role by linking predicted high-risk probabilities with uncertainty-aware selective prediction and case-level interpretation.

### 4.3. Limitations and Future Research

Several limitations should be noted. First, the study used a single public inspection archive from New York State, and the findings may not directly generalize to jurisdictions with different inspection rules, reporting practices, or enforcement environments. Second, the 2023–2025 window was selected to evaluate contemporary short-horizon temporal screening, but it does not capture all long-term regulatory trends or policy changes since the beginning of the archive. Third, the high-risk label was defined from inspection-recorded critical and unresolved critical violations; although this definition is operationally meaningful for inspection prioritization, it is not equivalent to laboratory-confirmed foodborne illness or outbreak occurrence. Fourth, first-visit establishments and sparse-history cases provide limited historical evidence, so predictions for these cases should be interpreted more cautiously. Fifth, the study was evaluated using retrospective temporal validation rather than prospective field deployment. Future work should test the framework in additional jurisdictions, longer time windows, and real inspection workflows, and should examine whether model-assisted prioritization improves inspection efficiency, high-risk case detection, and regulatory decision-making in practice.

### 4.4. Implications for Risk-Based Food Control and Conclusions

From a food control perspective, the most important implication of this study is that predictive inspection support should be viewed as a layered decision tool rather than as a replacement for official inspection judgment. Recent review work has emphasized that artificial intelligence, big data, and connected monitoring systems are reshaping food safety governance toward earlier, more predictive, and more adaptive forms of intervention [40,41]. Our results support this transition, but in a very specific way: the strongest gains emerged when historically grounded signals were combined with transparent model structure and actionable uncertainty estimates. This means that the most credible role for such models is to support triage, ranking, and prioritization before inspection, while preserving room for expert interpretation in borderline cases.

In practical inspection workflows, the proposed model is best understood as a pre-inspection decision-support tool rather than a replacement for inspector judgment. Its main benefits are threefold. First, it can rank upcoming inspection events according to predicted high-risk probability, helping agencies prioritize establishments with stronger historical risk signals when resources are limited. Second, because the model uses only pre-inspection metadata, historical numeric records, and prior comments, it can be applied before the current inspection outcome is known. Third, the evidential uncertainty score allows the system to distinguish high-confidence model-assisted prioritization from ambiguous cases that should remain under routine scheduling, manual review, or additional information collection. The quantitative improvements should be interpreted as retrospective test-set gains rather than as direct evidence that real-world inspection decisions have already improved by the same percentage. In the held-out test set, edl-mlp improved AUPRC from 0.398 for the strongest baseline catb to 0.424, a relative gain of approximately 6.5%, and improved F1 from 0.406 to 0.431, a relative gain of approximately 6.2%. Its ECE decreased from 0.025 to 0.012, corresponding to an approximately 52% relative reduction in calibration error. Under selective prediction, retained-set F1 increased from 0.431 at full coverage to 0.542 at 80% coverage, an approximately 25.8% relative increase within the retained high-confidence subset, with a 19.5% error reduction. These results indicate potential benefits for model-assisted screening and triage, while prospective field studies are still needed to quantify the actual improvement in inspector decisions during operational deployment.

Operationally, the model could be used at the inspection-planning stage. For upcoming or eligible inspections, agencies could input pre-inspection metadata, prior numeric inspection history, and previous inspection comments to obtain a predicted high-risk probability and an uncertainty score. High-probability and low-uncertainty cases could be prioritized for earlier review or targeted inspector briefing, whereas high-uncertainty cases would remain under routine scheduling, manual review, or additional information collection. The model therefore supports risk-based planning before the site visit, while official violation decisions remain based on on-site inspection and regulatory standards. Under the validation-selected threshold, edl-mlp detected 44.5% of true high-risk events in the held-out 2025 test set. With selective prediction, retained recall increased to 49.5% at 90% coverage and 55.8% at 80% coverage, indicating stronger detection within the retained higher-confidence subset. These values represent retrospective temporal-validation results rather than confirmed field-deployment detection rates; prospective implementation is needed to quantify actual operational improvement.

More broadly, our findings suggest that the future of data-driven food safety regulation will depend on the integration of three elements. The first is high-quality digital inspection history; the second is multimodal evidence fusion, especially the joint use of structured compliance records and prior narrative text; and the third is trustworthy uncertainty quantification that helps distinguish robust alerts from cases requiring additional review. These themes increasingly recur across recent discussions of AI-enabled food safety management [32,33,41]. Our empirical results give them a concrete regulatory form by showing that a leakage-free, temporally evaluated framework can convert archived inspection data into prospectively useful screening intelligence.

In conclusion, this study shows that high-risk food inspection events can be screened more effectively when prediction is grounded in prior establishment behavior, enriched by leakage-free historical narratives, and moderated by calibrated evidential uncertainty. The proposed EDL-MLP outperformed strong tabular baselines in discrimination, reliability, selective prediction, and interpretability, while preserving a modeling design that is consistent with real regulatory deployment. Taken together, these findings support a clear conclusion: the most promising path for AI-assisted food safety control is not simply to build more accurate classifiers, but to build temporally valid, multimodally informed, and uncertainty-aware systems that strengthen risk-based inspection practice.

## Figures and Tables

**Table 1:** Hyperparameter tuning ranges and final selected settings for the proposed EDL-MLP.

Component	Candidate Range or Rule	Final Setting
Historical text encoder	Fixed pretrained encoder	all-MiniLM-L6-v2; 384-dimensional output
Encoder fine-tuning	Frozen vs. fine-tuned	Frozen
Text projection dimension	{64,128,256}	128
Categorical embedding dimension	$d_{m}=\min(32,\max(4,\lceil 2|C_{m}{|}^{1/4}\rceil ))$	Cardinality-based rule
Hidden-layer widths	{(128,64),(256,128),(512,256),(256,128,64)}	(256,128)
Dropout rate	{0.10,0.20,0.30,0.50}	0.20
Optimizer	Adam, AdamW	AdamW
Learning rate	$\left\{5\times 10^{-4},1\times 10^{-3},2\times 10^{-3}\right\}$	$1\times 10^{-3}$
Weight decay	$\left\{0,1\times 10^{-5},1\times 10^{-4}\right\}$	$1\times 10^{-5}$
Training batch size	{128,256,512}	256
Validation/test batch size	Fixed for evaluation	512
Maximum epochs	{30,50}	30
Early-stopping patience	{5,6,10}	6 epochs
KL annealing horizon tann	{5,10,20}	10 epochs
Class-imbalance handling	None, inverse-frequency sampling	Inverse-frequency weighted mini-batch sampling
Model-selection metric	Validation AUROC, validation AUPRC	Validation AUPRC
Classification threshold	0.05–0.95 by 0.01	Validation F1-maximizing threshold

**Table 2:** Sample characteristics by temporal split.

Split	Inspections	Unique Ops	Positive Rate	First-Visit Rate	Re-Inspection Rate	Median Prior Inspections	Median Gap Days
Overall	55,454	20,082	7.97%	36.21%	14.46%	1.0	40.0
Train	32,444	16,979	8.12%	52.33%	14.86%	0.0	0.0
Valid	14,526	12,039	7.45%	16.06%	13.97%	1.0	212.5
Test	8484	7285	8.31%	9.08%	13.79%	2.0	147.0

Note: For first-visit inspection events, prior inspections and gap days were coded as 0 because no previous inspection record was available within the study window.

**Table 3:** Overall benchmark performance on the held-out test set.

Model	AUROC	AUPRC	Precision	Recall	F1	Brier	ECE
logit	0.742	0.231	0.254	0.291	0.271	0.074	0.045
rf	0.785	0.315	0.342	0.310	0.325	0.071	0.038
xgb	0.821	0.378	0.381	0.395	0.388	0.068	0.032
lgbm	0.825	0.385	0.386	0.402	0.394	0.067	0.030
catb	0.832	0.398	0.392	0.421	0.406	0.066	0.025
mlp	0.795	0.332	0.335	0.380	0.356	0.072	0.042
ftt	0.812	0.365	0.368	0.405	0.386	0.069	0.035
tabm	0.830	0.395	0.390	0.418	0.404	0.066	0.028
edl-mlp	0.846	0.424	0.418	0.445	0.431	0.063	0.012

Note: AUROC ranges from 0.5 to 1.0 for practical discrimination, where 0.5 indicates chance-level ranking and values closer to 1.0 indicate stronger discrimination. AUPRC, precision, recall, and F1 range from 0 to 1, with values closer to 1.0 indicating better screening performance; AUPRC should be interpreted relative to the positive-class prevalence. Brier score and ECE range from 0 to 1, with values closer to 0 indicating better probabilistic accuracy and calibration.

**Table 4:** Ablation results of the proposed framework on the held-out test set.

Variant	AUPRC	F1	Brier	ECE	Δ AUPRC vs. Full
Full model	0.424	0.431	0.063	0.012	—
w/o evidential head	0.408	0.415	0.067	0.041	−0.016
w/o recent rolling features	0.415	0.422	0.064	0.014	−0.009
w/o historical text	0.392	0.405	0.066	0.017	−0.032
w/o historical numeric block	0.318	0.345	0.071	0.025	−0.106
Metadata-only	0.245	0.280	0.075	0.038	−0.179

Note: AUPRC and F1 range from 0 to 1, with values closer to 1.0 indicating better high-risk screening performance. Brier score and ECE range from 0 to 1, with values closer to 0 indicating better probabilistic reliability and calibration. Δ AUPRC is calculated relative to the full model; values close to 0 indicate little loss relative to the full model, whereas more negative values indicate a larger reduction in screening performance after removing the corresponding component.

**Table 5:** Selective-prediction performance at fixed coverage levels.

Model	F1 (100%)	F1@90%	Retained Recall@90%	F1@80%	Retained Recall@80%	Error Reduction@80%
edl-mlp	0.431	0.486	0.495	0.542	0.558	19.5%
catb	0.406	0.435	0.448	0.471	0.485	10.9%
tabm	0.404	0.428	0.440	0.458	0.466	9.1%

Note: Coverage denotes the target proportion of cases retained for model-assisted prediction after excluding the most uncertain cases according to the validation-derived uncertainty threshold. Deferral does not imply that cases are ignored or not inspected; rather, deferred cases are routed to routine scheduling, manual review, or additional information collection. Retained recall is calculated using only the predictions retained at the target coverage level, rather than all positive cases in the full test set.

## Data Availability

The raw data used in this study are publicly available from Health Data NY as “Food Service Establishment Inspections: Beginning 2005 (ACTIVE)” at https://health.data.ny.gov/ (accessed on 10 March 2026). The open-source implementation of the proposed model is available at https://github.com/caymarheng/edl-mlp (accessed on 10 March 2026).
